# Importance of the gut microbiota in mice with a ‘humanized’ bile acid pool

**DOI:** 10.1042/CS20231465

**Published:** 2024-01-10

**Authors:** Justine Gillard, Isabelle A. Leclercq

**Affiliations:** Laboratory of Hepato‐Gastroenterology, Institute of Experimental and Clinical Research, Université catholique de Louvain, Brussels, Belgium

**Keywords:** Bile acids, Cyp2c70, Gut microbiota, Hepatobiliary disease, Hydrophobicity, UDCA

## Abstract

Bile acids are signaling mediators, enabling intricate communication between tissues and the gut microbiota, and are involved in the pathophysiology of several immune and metabolic disorders. In this commentary, we discuss the importance of the gut microbiota in the *Cyp2c70* knock-out mice, which are considered as a promising ‘humanized’ experimental resource for studying bile acids and their role in pathological conditions. We also discuss how *Cyp2c70*-deficient mice contribute to enhancing the translatability of preclinical studies in murine models to humans.

Mice are commonly used as experimental models to mimic and study human pathologies. However, when investigating conditions related to bile acids, conventional mice may not be the optimal choice, due to species-specific differences (recently reviewed in [1]). Bile acid metabolism, including interactions between host and gut microbiota, significantly differs between humans and mice. In humans, the bile acid pool predominantly contains cholic (CA), chenodeoxycholic (CDCA), and deoxycholic (DCA) acids, whereas muricholic acids (MCAs) account for a large proportion of the bile acid pool in mice (Figure 1). The production of 6β-hydroxylated MCAs by murine livers has been documented for decades; however, the identification of CYP2C70, the enzyme responsible for the hydroxylation of bile acids at the 6β position (as schematically depicted in Figure 1), was only achieved in 2016 by Takahashi and colleagues [2]. Expanding on this significant advancement, Folkert Kuipers’ group successfully generated hepatic *Cyp2c70* knock-out mice by employing the CRISPR/Cas9 technology [3]. They documented a drastic reduction of MCAs and an accumulation of CDCA in the bile acid pool of mice lacking *Cyp2c70* (Figure 1) [3]. Subsequently, several independent research groups swiftly confirmed these initial observations, illustrating that *Cyp2c70*-deficient mice exhibited a bile acid pool free of MCAs, resembling that of humans [4,5]. Therefore, *Cyp2c70* knock-out mice were considered as a promising ‘humanized’ experimental resource for investigating bile acids and their role in pathological contexts, for developing and testing potential treatments, and for significantly enhancing the translatability of findings from mice to humans.

The hydroxylation at the 6β position significantly impacts on the physicochemical properties of bile acids. Having an additional hydroxyl group, MCAs are inherently more hydrophilic compared with CDCA. Consequently, the substantial increase of CDCA within the bile acid pool of *Cyp2c70*-deficient mice results in a more hydrophobic and therefore potentially more toxic bile acid pool (Figure 1). Indeed, mice lacking *Cyp2c70* have liver damage (Figure 1). Neonatal cholestasis has been documented, accompanied by elevated transaminases, cholangiocyte proliferation, and a pro-inflammatory and pro-fibrotic gene signature not seen in controls [6]. As *Cyp2c70*-deficient mice age, liver damage progresses with ductular reaction, hepatocyte necrosis, lymphocytes and neutrophils infiltration ultimately evolving to fibrosis in the livers of adult *Cyp2c70*-deficient mice [4,6]. However, the mechanisms by which bile acids cause damage to liver cells and initiate the inflammatory response in *Cyp2c70*-deficient mice remain undetermined. Additionally, it is noteworthy that the *Cyp2c70* deletion induces a more pronounced liver phenotype in females compared with males [6]. The spontaneous hepatobiliary injuries observed in *Cyp2c70*-deficient mice thus raise questions about their suitability to study pathophysiological processes involved in liver diseases.

**Figure 1:**
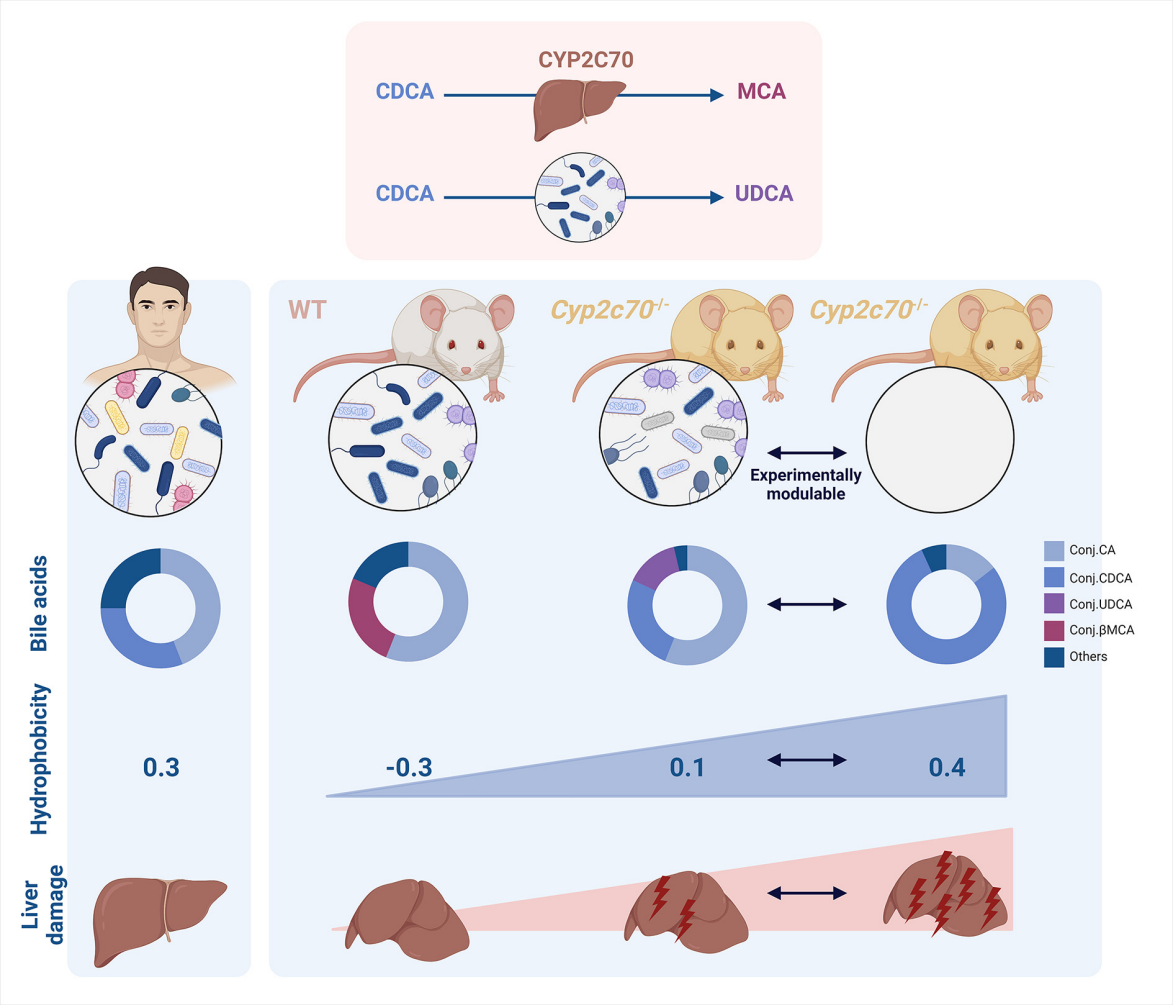
Effects of *Cyp2c70* deletion and gut microbiota depletion on the composition and hydrophobicity of the bile acid pool and liver damage. The upper panel illustrates the simplified conversion of CDCA to αMCA and βMCA by CYP2C70 in mouse livers, and the conversion of CDCA to UDCA by gut bacterial enzymes. In the lower panels, the composition and hydrophobicity of gallbladder bile, as well as liver damage, are depicted in humans, WT mice, and *Cyp2c70*-deficient mice with and without a gut microbiota. Bile acids are conjugated to taurine in mice, whereas they are conjugated to both taurine and glycine in humans. Human data is sourced from [11], and mouse data from [8,9].

In addition to liver enzymes, bacterial enzymes also shape the composition and the physiochemical properties of the bile acid pool [7]. Bile salt hydrolases remove glycine and taurine conjugates from bile acids. Enzymes for 7α-dehydroxylation pathway convert CA and CDCA to DCA and lithocholic acid (LCA), respectively. Additionally, CDCA can be epimerized by hydroxysteroid dehydrogenases to form ursodeoxycholic acid (UDCA). Deconjugation and dehydroxylation carried by gut bacteria increase the hydrophobicity index of bile acids, while epimerization of CDCA to UDCA reduces it. In turn, bile acids shape the gut microbial composition by exerting bacteriostatic and bactericidal effects. To illustrate, *Cyp2c70*-deficient mice exhibit a different microbial composition in their caeca compared with wild-type mice, with over 40 genera showing differential abundance [6].

In two recent research papers published in *Clinical Science*, the interaction between bile acids and the gut microbiota was investigated to gain a deeper understanding of the mechanisms of hepatobiliary injuries observed in *Cyp2c70*-deficient mice. The approach consisted of depleting the gut microbiota. In a first study by Sjöland and colleagues, *Cyp2c70*-deficient mice were rederived as germ-free, and were then colonized (or not) with human or mouse gut microbial communities [8]. In a second study by Verkade et al., *Cyp2c70*-deficient mice received broad-spectrum antibiotics to eliminate their gut microbiota [9]. The bile acid pool of germ-free *Cyp2c70*-deficient mice was composed of TCDCA and TCA (Figure 1), in line with the absence of *Cyp2c70* to convert CDCA to MCAs and with the absence of bacterial enzymes to deconjugate bile acids and convert them to UDCA, DCA or LCA [8]. Colonization of germ-free *Cyp2c70*-deficient mice with a human gut microbiota did not significantly change the bile acid pool. By contrast, colonization with a mouse gut microbiota massively increased the proportion of UDCA, as a result of the conversion of CDCA to UDCA by bacterial enzymes [8]. The increase in UDCA (and conjugated forms) at the expense of CDCA resulted in a less hydrophobic bile acid pool [8]. In the study by Verkade et al., gut microbiota depletion resulted in a bile acid pool composed mainly of TCDCA in *Cyp2c70*-deficient mice (Figure 1) [9]. The cumulated absence of hepatic *Cyp2c70* and bacterial 7α/β-hydroxysteroid dehydrogenase prevent the conversion of CDCA to MCAs and to UDCA [9]. Moreover, the repression of hepatic CYP8B1 compromised the production of 12α-hydroxylated CA, tilting the balance towards the production of non 12α-hydroxylated CDCA in livers of antibiotic-treated *Cyp2c70*-deficient mice [9]. In the two studies, the combination of *Cyp2c70* deletion and gut microbiota depletion massively increased the proportion of TCDCA - up to 90% - and thus, the hydrophobicity of the bile acid pool (Figure 1).

The changes in bile acid pool hydrophobicity had a noticeable effect on the mouse phenotype. Germ-free *Cyp2c70*-deficient mice had enlarged livers, with significant liver fibrosis and cholangiocyte proliferation compared with germ-free WT mice [8]. High mortality in young germ-free *Cyp2c70*-deficient mice was reduced when mice were colonized with human or mouse gut microbial communities [8]. In line with decreased hydrophobicity of the bile acid pool, the colonization of *Cyp2c70*-deficient mice with a mouse, but not with a human, gut microbiota completely resolved fibrosis back to normal in adult mice [8]. In the study by Verkade et al., treatment of mice lacking *Cyp2c70* with antibiotics, resulting in a highly hydrophobic bile acid pool, exacerbated inflammation, fibrogenesis and ductular reaction [9]. In the specific context of *Cyp2c70* deletion, the depletion of gut bacteria even compromised survival of the animals, highlighting the severity of the hepatobiliary damage.

The notion that a hydrophobic bile acid pool contributes to liver injury received further validation through two independent experiments conducted by Verkade and colleagues. In one experiment, they reduced the production of 12α-hydroxylated bile acids by knocking down *Cyp8b1* in *Cyp2c70*-deficient mice, promoting the production of CDCA [9]. This manipulation increased the hydrophobicity of the bile acid pool and promoted hepatobiliary damage [9]. Conversely, in a distinct experiment, antibiotic-treated *Cyp2c70*-deficient mice were fed a diet containing UDCA, which lowered the hydrophobicity of the bile acid pool, lessening liver injury [9]. These findings are in line with two other studies conducted by the group of Folkert Kuipers and Jan Freark de Boer, in which UDCA was shown to reverse cholangiopathy and improve liver function in young *Cyp2c70*-deficient mice [6,10]. Collectively, these studies highlight (i) the importance of regulating the hydrophobicity of the bile acid pool to maintain liver homeostasis and prevent toxicity, and (ii) the role played by the gut microbiota in adjusting the composition and physicochemical properties of the bile acid pool.

These two studies undeniably enhance our understanding of the pathological changes that take place in *Cyp2c70*-deficient mice with a so-called ‘humanized’ bile acid liver metabolism. They also emphasize the importance of the gut microbiota and its capacity to produce (T)UDCA, constraining the hydrophobicity of the bile acid pool. Nevertheless, despite this significant progress, numerous substantial questions still await answers. It may be necessary to ‘humanize’ the gut microbiota of *Cyp2c70*-deficient mice to enhance the translational relevance of this model. However, colonization of mice lacking *Cyp2c70* with a human microbiota was less efficient in alleviating liver pathology than their colonization with a mouse microbiota. The reasons why human and mouse microbiota result in divergent responses deserve further investigations. Notably, it would be of great interest to pinpoint the specific bacteria, bacterial enzymes or bacterial metabolites responsible for the divergences. Besides, host factors such as immune system for defense against microbes, bile acid transport system or response to bile acid receptor activation may also be at play. Identifying these factors would establish a causality, advancing the research beyond mere associations. As a future application, targeting bile acid metabolism in the gut could be considered for therapy in patients with cholestatic liver diseases.

## Data Availability

N/A

## Abbreviations

CA: cholic acid
CDCA: chenodeoxycholic
DCA: deoxycholic
LCA: lithocholic acid
MCA: muricholic acid
UDCA: ursodeoxycholic acid
